# Health related quality of life among hypertensive adults living in rural Vietnam: Results from a cluster-randomised controlled trial

**DOI:** 10.1371/journal.pone.0337817

**Published:** 2026-05-13

**Authors:** Cuc T. T. Nguyen, Jahnavi Challagonda, Tung S. Ha, Nhi D. Nguyen, Huong T. T. Nguyen, Duc A. Ha, Oanh T. Tran, Phuong K. Nguyen, Van H. Phan, Giang H. Nguyen, Thu H. T. Nguyen, Robert J. Goldberg, Jeroan J. Allison, Bo Wang, Germán Chiriboga, Hoa L. Nguyen

**Affiliations:** 1 Health Strategy and Policy Institute, Hanoi, Vietnam; 2 Hanoi Medical University, Hanoi, Vietnam; 3 UMass Chan Medical School, Worcester, Massachusetts, United States of America; 4 Division of Genomic Medicine, Vinmec Hi-Tech Center, Vinmec Healthcare System, Hanoi, Vietnam; 5 Donald and Barbara Zucker School of Medicine at Hofstra/Northwell, Hempstead, New York, United States of America; 6 Department of Geriatrics, Hanoi Medical University, Hanoi, Vietnam; 7 Scientific Research Department, National Geriatric Hospital, Hanoi, Vietnam; 8 Vietnam Ministry of Health, Hanoi, Vietnam; 9 Department of Health Management and Organization, School of Preventive Medicine and Public Health, Hanoi Medical University, Hanoi, Vietnam; 10 Department of Population and Quantitative Health Sciences, UMass Chan Medical School, Worcester, Massachusetts, United States of America; International Medical University, MALAYSIA

## Abstract

**Background:**

There is a growing burden of hypertension (HTN) among adults living in rural Vietnam, which is associated with reduced health related quality of life (HRQoL). Few community-based interventions have, however, attempted to improve the quality of life in patients with uncontrolled HTN. This study aimed to examine the impact of a multi-component intervention on HRQoL in adults with uncontrolled HTN.

**Methods:**

This cluster-randomized controlled trial was conducted in sixteen communities (8 intervention and 8 comparison) living in a rural setting in Vietnam (2017−2022). Consenting adults with uncontrolled HTN were enrolled. The comparison arm received training sessions about HTN prevention and management and patient education materials. The intervention arm received information similar to the comparison group and three enhancement components, namely a storytelling intervention, home blood pressure (BP) self-monitoring, and expanded community health worker services. The primary outcome was the differential change in HRQoL over 12-month follow-up period, measured using the 12-Item Short Form Health Survey (SF-12), which generates a Physical Component Summary (PCS-12) and a Mental Component Summary (MCS-12). The total HRQoL score was calculated as the sum of PCS-12 and MCS-12, with higher scores indicating better HRQoL.

**Results:**

A total of 671 patients were studied; their mean age was 66 years and 55% were women. At the 12-month follow-up, the intervention group showed a significant increase in their PCS-12 with a multivariable-adjusted difference of 4.2 points (95% CI: 2.0–6.4) compared with the control group. While the MCS-12 scores increased for both groups, their differential change over 12 months was not statistically significant (multivariable-adjusted difference: 1.4 points; 95% CI: −0.6; 3.5).

**Conclusion:**

Our results demonstrate that a multicomponent intervention effectively improved overall HRQoL with a significant impact on physical health-related HRQoL in individuals with uncontrolled HTN.

**Trial registration:**

ClinicalTrials.gov, Registration number: https://clinicaltrials.gov/study/NCT03590691, (registration date July 17, 2018)

## Introduction

Non-communicable diseases are a significant public health challenge globally, with cardiovascular diseases representing one of the leading causes of morbidity and mortality, particularly in low- and middle-income countries, such as Vietnam. Based on the 2021 National Noncommunicable Disease Risk-Factor Surveillance surveys, approximately 28% of Vietnamese adults aged 25–64 years were characterized as having hypertension [1]. This significant prevalence poses major complications for public health systems and patients’ quality of life [2].

Health Related Quality of life (HRQoL) is an important indicator for evaluating treatment outcomes and assessing the overall effectiveness of different management strategies for adults with hypertension (HTN). Recent research has shown that factors such as medication adherence, lifestyle modifications, and social support networks significantly influence HRQoL trajectories among individuals with HTN [3]. Additionally, evidence from previous international studies has shown that HTN is linked to decreased HRQoL [4–7].

Despite increasing recognition of HTN’s negative impact on HRQoL, there are limited studies that have examined HRQoL among patients with HTN in Vietnam, particularly among those living in rural settings. Previous studies have found that patients with HTN in Vietnam generally reported moderate levels of HRQoL [8–10], with those residing in rural areas experiencing significantly lower HRQoL, as compared to persons without HTN. To the best of our knowledge, there is a lack of published community-based interventions aimed at improving HRQoL for patients with HTN in rural Vietnam.

The *Conquering Hypertension in Vietnam: Solutions at Grassroots level* study (the parent study) was a cluster-randomized controlled clinical trial conducted in 16 rural communities in Hung Yen province, Northern Vietnam. The study evaluated the effectiveness of a multi-component intervention to improve blood pressure (BP) control and various secondary outcomes among patients with uncontrolled HTN [11,12]. The intervention included three modalities: storytelling, home blood pressure monitoring, and expansion of community health workers. In the present study, we examined the impact of this multi-faceted intervention on HRQoL (a secondary trial outcome) among patients with uncontrolled HTN residing in rural Vietnam using data from the parent study.

## Methods

### Study design and setting

This study was a cluster-randomized controlled trial designed to evaluate the effectiveness of a multicomponent intervention on blood pressure control and HRQoL among adults with uncontrolled hypertension. The detailed study design has been published elsewhere [11–13]. The study was conducted in 16 communities in northern Hung Yen province, Vietnam, with 8 communities assigned to the intervention group and 8 communities to the control group. Patients were enrolled at their local community health center and followed up at 12 months after randomization. The protocol for this trial has been previously published [11], and the supporting CONSORT checklist is included in Supporting information (S1 File). The trial was registered at ClinicalTrials.gov (Registration number: NCT03590691, registration date July 17, 2018)

### Patient eligibility

Potentially eligible participants were adults aged 25 years and older living in selected rural communities, without plans to relocate within 12 months, and who met the criteria for uncontrolled HTN as defined by the 8^th^ Joint National Commission of High Blood Pressure guidelines (JNC 8) [14]. These patients had to have no cognitive impairment, not be pregnant, and not be involved as storytellers in the parent trial intervention. Participant screening was conducted at local community health centers by trained health personnel. Screening for the parent study required two blood pressure (BP) measurements taken at least one week apart, following a standardized protocol. Individuals with persistently elevated BP—defined as >140/90 mmHg at both screening visits—were considered eligible for enrollment. Both incident (newly diagnosed) and prevalent cases of uncontrolled HTN were enrolled in the parent trial. Newly diagnosed patients were referred for antihypertensive initiation, while those with severe BP (BP greater than 180/110 mmHg) were referred for treatment adjustment. Written informed consent was obtained from all trial participants prior to study enrollment.

### Study recruitment, randomization, and masking

This cluster-randomized controlled trial was conducted in 16 communities in four districts in Hung Yen Province, Vietnam. Randomization was performed at the community level, stratified by district, using a computer-generated random number sequence. In each district, two communities were allocated to the intervention arm and two to the comparison arm. Eligible consenting adults with uncontrolled HTN were assigned to either of these groups based on their residence. Both study patients and those assessing the primary and secondary trial outcomes were blinded to treatment allocation to minimize the potential for measurement bias. Recruitment occurred from October 2019 to April 2021.

### Intervention content and delivery

#### Vietnam National Hypertension Program (Both groups; provided at community level).

Both the intervention and comparison groups received the Vietnam National Hypertension Program [15]. This program provided physician and nursing training on HTN prevention, diagnosis, treatment, and patient counseling, along with educational materials for patients. There were two training sessions (September 2019 and July 2020) that were held for 54 clinicians to update their knowledge and skills for diagnosing and managing patients with HTN. Posters and leaflets promoting HTN control were distributed to patients at the local community health centers to increase their awareness of how to best improve the control of their HTN.

#### Storytelling intervention (Intervention group only; provided at patient level).

In the past, we developed and pilot-tested a storytelling intervention for the control of HTN in Vietnam featuring patients sharing their personal experiences about managing BP, supplemented by additional information about how to best control their BP through a Didactic Learn More section [16,17]. Four DVDs were created and delivered to intervention participants. The first DVD was introduced at enrollment at the local community health center, while the remaining DVDs were provided at the clinic follow-up visits or delivered to participants’ home. These DVDs were given at baseline and at three, six-, and nine-months post-enrollment. In contrast, the comparison group received two DVDs containing only didactic content focus on HTN management, including advice about dietary recommendations, smoking cessation, alcohol reduction, and importance of participation in physical activity. These approaches were provided at baseline and at a six-month study visit, along with a DVD player.

#### Home blood pressure self-monitoring (Intervention group only; provided at patient level).

Patients in the intervention group received calibrated Omron BP monitors at the time of trial enrollment. They were given instructions from community health workers on its proper use and logging of BP readings. Participants were instructed to measure their BP twice daily—once in the morning and once in the evening—during the study period.

#### Expanded community health worker services (Intervention group only; provided at community and patient levels).

The role of the community health workers was strengthened through the implementation of the intervention since they promoted lifestyle changes and importance of medication adherence to enhance BP control. In collaboration with the Vietnam Ministry of Health, two training sessions were held for 41 CHWs from the eight intervention communities. These sessions focused on motivational interviewing strategies, problem-solving techniques, and how to use the storytelling DVDs to guide patient discussions. After each DVD, the CHWs met with patients to address any barriers the participants faced with making lifestyle behavior changes while working with them to develop new strategies to better control their elevated BP. In addition, CHWs provided bi-weekly home visits to support DVD viewing and BP monitor use.

### Follow-up activities

After obtaining informed consent and trial enrollment, study participants received reminder letters before each of three follow-up visits (3-, 6- and 12 months after trial enrollment) with phone calls one week prior. If participants missed any in-person clinic visits, this led to follow-up phone calls or home visits for measurement of their BP. This study was paused twice during the COVID-19 pandemic (February – April 2021 and July-October 2021) which led to substantial missing data at the three- and six-month follow-ups. Therefore, only data from the study baseline period and the 12- month follow-up were included in the present study.

### Sample size

Detailed sample size calculations for this trial have been previously described [11].

### Data collection

Trained healthcare professionals conducted interviews to gather data on patient’s socio-demographic characteristics, medical history, lifestyle factors using the validated STEP survey [18]. Age (years) was treated as a continuous variable. Other variables were categorized using a similar approach with the previous publication [12] including sex (male vs. female), education level (less than primary, primary, secondary, high school, and college/university), marital status (currently married vs. other), and occupation (employed, self-employed, farmer, retired, homemaker, or other), smoking status (self-reported current smoker vs. non-smoker). Anthropometric measurements, including height, weight, waist circumference, and hip circumference, were taken using standardized equipment and protocols. Body Mass Index (BMI) was calculated and treated as a continuous variable.

BP was measured using a validated automated device. Three BP readings were taken by trained health personnel, with participants seated and rested, following the World Health Organization standard protocol [19]. The average of the last two measurements was used in subsequent analyses. HRQoL scores were measured using SF-12 survey instrument [20].

### Measurement of quality of life

HRQoL was defined as the patient’s overall well-being, which was dependent on three independent factors including objective descriptors, subjective evaluations, and personal values and aspirations. These parameters were based on physical, mental, emotional, social, and material wellbeing [21]. HRQoL was assessed using the 12-Item Short Form Health Survey (SF-12), a validated and reliable instrument for measuring health-related HRQoL across diverse populations, including Vietnam. The SF-12 survey was constructed using questions drawn from the Medical Outcomes Study 36-item Short Form Survey (SF-36), which was designed to have similar performance to the SF-36, while taking less time to complete [20,22,23].

The SF-12 focuses on two main components: the Physical Component Summary (PCS-12) and the Mental Component Summary (MCS-12). It covers eight health domains: physical functioning, role physical, bodily pain, general health, vitality, social functioning, role emotional, and mental health. These domains are weighted and combined to calculate the PCS-12 and MCS-12 scores, which represent physical and mental health, respectively.

The calculation of PCS-12 and MCS-12 scores followed the standard published algorithm [20]. Each of the 12 items in the SF-12 was first recoded and normalized based on the scoring guidelines. Responses to each item were assigned specific weights, which were derived from population norms. These weights were used to calculate raw scores for the physical and mental health components.

Recoding Responses: The questions in the SF-12 use various Likert scales, such as three levels (yes, very limited; yes, limited; not limited), five levels (no influence; little influence; moderate influence; lot of influence; very much influence), six levels (always; most of the time; quite a lot; occasionally; sometimes; none of the time), two levels (yes, no), and health rating questions (excellent, very good, good, fair, poor). The scoring method produces scores for each component (PCS and MCS) ranging from 0 to 100 (50 is considered average). The total HRQoL score was calculated as the sum of the physical health and mental health component scores. Higher scores indicate better HRQoL; items related to limitations in physical functioning or emotional problems were reverse coded so that a higher score indicated fewer limitations.

Applying Weights: The recoded responses were multiplied by their respective weights for PCS-12 and MCS-12. These weights were derived from a U.S. general population reference and were specific to each item. For example, items related to physical functioning contributed more to the PCS-12 score, while items related to mental health contributed more to the MCS-12 score.

### Study outcome

The primary outcome of interest was the change in HRQoL (the PCS-12 and the MCS-12) over a 12-month period (continuous outcomes), assessed at the individual patient level. The main analysis focused on comparing the differential change in HRQoL between participants in the intervention and comparison groups from baseline to the 12-month follow-up.

### Data analysis

Patient’s baseline characteristics were compared between the intervention and control groups. Categorical variables were presented as percentages and compared using chi-square tests, while continuous variables were presented as means with standard deviations and compared using t-tests. Each patient contributed data at two time points (baseline and 12-month follow-up).

We examined differences in over-time changes in HRQoL between the two primary study groups using linear mixed models, including study group (intervention vs. comparison), time (baseline vs. 12 months), and the interaction term between study group and time. This was done to estimate the differential intervention effect over time, accounting for clustering of patients within the community and repeated within-person measurements by random effects. We also used the mixed models to adjust for potential covariate imbalances between the two groups at baseline, including age, sex, education level, marital status, BMI, smoking status, and baseline anti-hypertensive medication use. Additionally, we performed subgroup analyses according to sex, age group, and education level to explore the impact of the study intervention across different subgroups. All analyses were performed using STATA 17.0 statistical software (USA).

### Ethical considerations

This randomized trial was approved by the Institutional Review Board at the Health Strategy and Policy Institute (HSPI) in Hanoi, Vietnam (Decision 171/QD-CLCSYT dated September 10, 2019). Written informed consent was obtained from all patients.

## Results

**Fig 1:**
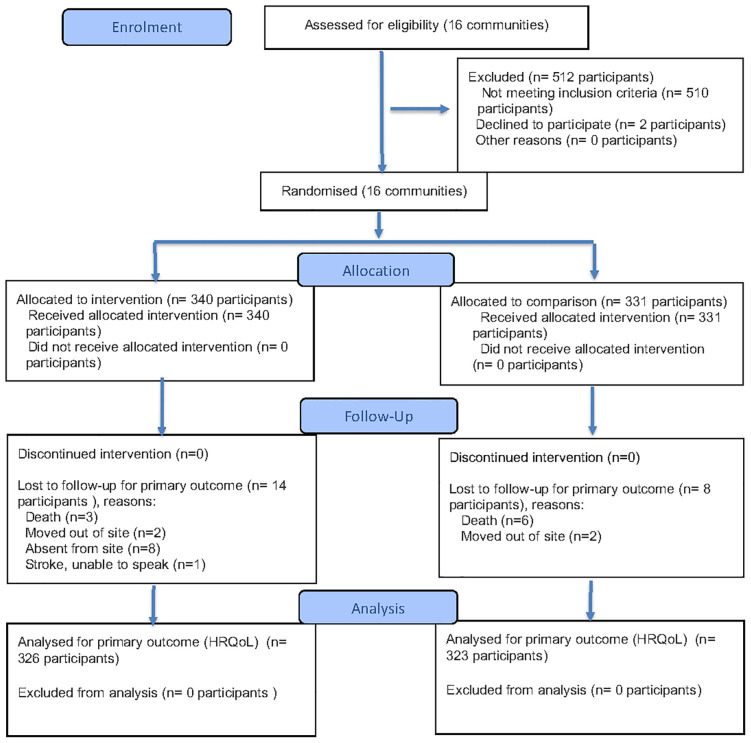
Flow diagram of the cluster randomized controlled trial.

A total of 671 participants with uncontrolled HTN from 16 communities (8 intervention, 8 comparison) in rural Northern Vietnam were enrolled in the study, including 340 participants in the intervention group and 331 in the comparison group. At the 12-month follow-up, data were available for 97% of participants (649 of 671) (Fig 1). At baseline, the mean PCS-12, MCS-12, and total HRQoL scores for the entire study population were 41.8, 51.9, and 93.7, respectively.

### Baseline patient characteristics by study group

The intervention and comparison groups were generally comparable in terms of their demographic and clinical characteristics, but some differences were observed (Table 1). Patients in the intervention group were slightly younger, had a lower body mass index, and had a higher proportion of males and participants who were currently married compared with those randomly assigned to the comparison group. The intervention group had a higher proportion of participants with a high school or higher education at baseline compared to the comparison group.

**Table 1:** Baseline characteristics of study participants by study groups.

	Intervention (n = 340)	Comparison (n = 331)
Male (n,%)	161 (47.4)	141 (42.6)
Age (mean, SD, range), years	65.7 (8.9, [38-84])	67.9 (9.0, [42-91])
Marital status (n,%)		
Currently married	277 (81.5)	253 (76.4)
Other	63 (18.5)	78 (23.6)
Level of Education (n,%)		
Primary school and lower	73 (21.5)	100 (30.2)
Secondary school	190 (55.9)	164 (49.6)
High school and upper	77 (22.6)	67 (20.2)
Work status (n,%)		
Currently working	151 (44.4)	142 (42.9)
Retired	78 (22.9)	63 (19.0)
Homemaker	39 (11.5)	41 (12.4)
Other	72 (21.2)	85 (25.7)
Body mass index (mean, SD), kg/m^2^	22.9 (3.0)	23.1 (2.7)
Ever smoked (n,%)	105 (35.1)	93 (31.4)
HRQoL score (mean, SD)		
PCS-12	41.3 (0.6)	42.3 (0.6)
MCS-12	52.6 (0.5)	51.1 (0.5)
Total score	93.9 (0.8)	93.4 (0.8)
SD: Standard deviation; BMI: Body Mass Index		

### Baseline HRQoL according to study groups

**Fig 2:**
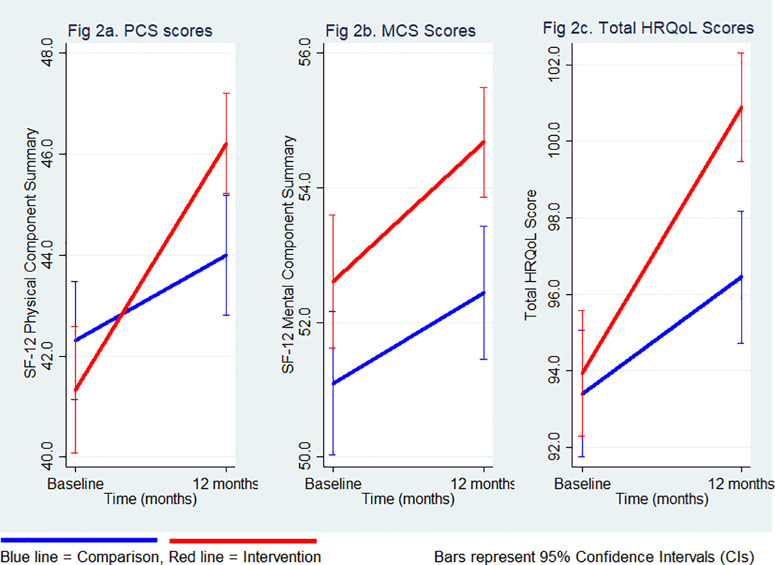
Changes in PCS-12, MCS-12, and total HRQoL scores (95% CI) according to study groups.

At the baseline, there was no significant difference in total HRQoL scores between the intervention and comparison groups (Table 2). When examining each component of the HRQoL, the mean PCS-12 score was comparable between the intervention and comparison groups (41.3 vs. 42.3) (Table 2 and Fig 2a). Similarly, no significant difference in the mean MCS-12 scores was observed between the two groups at the baseline (52.6 vs. 51.1) (Table 2 and Fig 2b).

**Table 2:** Differential over-time change for intervention vs. comparison groups (95% CIs) in health related quality of life.

	Intervention	Comparison	Difference over time in HRQoL scores	
			Unadjusted beta coefficient for interaction term (95% CI)	Adjusted beta coefficient for interaction term (95% CI)
**Number of communities**	**8**	**8**		
**Number of patients**	**326**	**323**		
PCS-12 (mean, SD)				
Baseline	41.3 (0.6)	42.3 (0.6)		
12 Months	46.2 (0.5)	44.0 (0.6)		
Δ Baseline- 12 Months	4.7 (0.7)	1.8 (0.7)	**3.1 (1.2–4.9)**	**4.2 (2.0–6.4)**
MCS-12 (mean, SD)				
Baseline	52.6 (0.5)	51.1 (0.5)		
12 Months	54.7 (0.4)	52.4 (0.5)		
Δ Baseline- 12 Months	2.1 (0.6)	1.3 (0.7)	0.8 (−1.0; 2.5)	1.4 (−0.6; 3.5)
Total Score (mean, SD)				
Baseline	93.9 (0.8)	93.4 (0.8)		
12 Months	100.9 (0.7)	96.4 (0.9)		
Δ Baseline- 12 Months	6.9 (0.9)	3.1 (1.0)	**3.9 (1.3–6.5)**	**5.6 (2.6–8.6)**
SD: Standard deviation				

*Results from linear models including study group, time, and interaction terms between the study group and time, accounting for clustering patients within a community and repeated measures nested within patients. The models adjusted for age, sex, educational level, marital status, smoking status, BMI, and baseline anti-hypertensive medication use. Intra-cluster correlation coefficients (ICCs) for PCS-12 were 0.03 and 0.37 for community and both (community and patient level), respectively. ICCs for MCS-12 were 0.02 and 0.18 for community and both (community and patient level), respectively. ICCs for Total HRQoL *score were 0.18 and 0.35 for community and both (community and patient level), respectively*.

### Changes in HRQoL over time according to study groups

Both study groups demonstrated improvements in their total HRQoL scores over the 12-month follow-up period; however, a greater increase was observed for patients in the intervention group compared with those in the comparison group (mean change: 6.9 points vs. 3.1 points), with a multivariable adjusted between-group difference of 5.6 points (95% CI: 2.6–8.6; p-value for the interaction term between study group and time <0.001) (Table 2 and Fig 2c).

In examining each component of HRQoL over the 12-month follow-up, the intervention group showed a significant increase in the PCS-12 compared to the comparison group (mean change: 4.7 points vs. 1.8 points), with a multivariable-adjusted difference of 4.2 points (95% CI: 2.0–6.4, Table 2 and Fig 2a). Improvements in the MCS-12 were more modest in both groups (mean change 2.1 points in the intervention group vs 1.3 points in the comparison group) with the adjusted between-group difference being 1.4 points (95% CI: −0.6; 3.5, Table 2 and Fig 2b).

### Subgroup analyses

**Fig 3:**
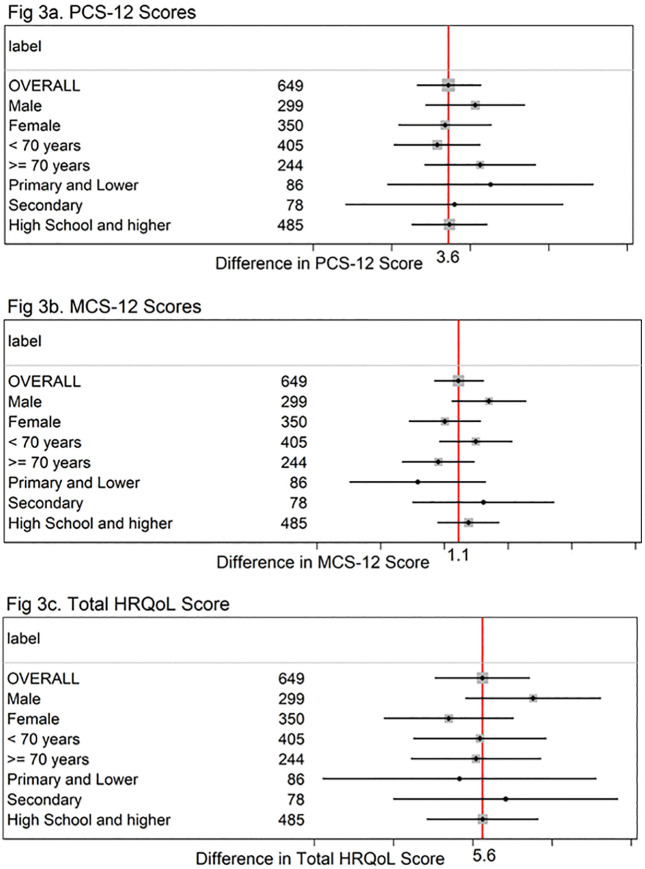
Changes in PCS-12, MCS-12, and total HRQoL from Baseline to 12 months by study groups.

In examining the possible heterogeneity of the intervention effects, we conducted several pre-specified subgroup analyses to assess whether the impact on total HRQoL, PCS-12, and MCS-12 scores differed across various patient characteristics at the time of 12-month follow-up. The Forest plots suggest that there is no evidence of heterogeneity of the multi-component trial intervention (Figs 3a, 3b, 3c).

### Serious Adverse Events (SAEs)

During the course of the trial, a total of three participants (0.9%) in the intervention group and six participants (1.8%) in the comparison group died. In addition, 27 participants (7.9%) in the intervention group and 9 participants (2.7%) in the comparison group were hospitalized for various conditions, including stroke, pleural effusion, diabetes, lung cancer, and renal failure.

## Discussion

This cluster randomized controlled trial of nearly 700 patients found that patients with uncontrolled HTN who were living in rural Vietnam had a low average PCS-12, but a slightly above-average MCS-12 score. Our multicomponent intervention significantly improved overall HRQoL, with greater gains observed in the intervention compared with the comparison group. Notably, significant improvements in the PCS-12 score were observed in the intervention compared with the control group over our 12-month follow-up period, while changes in the MCS-12 were less pronounced and not significant between the intervention and comparison groups. There is no evidence of heterogeneity of the multi-component trial intervention. The intervention was not associated with any adverse outcomes in trial participants.

### Impact of the multi-component study intervention on HRQoL

Our community-based intervention showed significantly improved overall HRQoL, particularly in the PCS, in our study sample of adults with uncontrolled HTN. These results are consistent with earlier work in low- and middle-income countries [24,25]. For example, a cluster-randomized controlled trial conducted among more than 2,600 adults with HTN in rural communities across Bangladesh, Pakistan, and Sri Lanka (2016−2018) evaluated the impact of a five-component community-based intervention (BP monitoring, home health education, physician training, HTN care coordinators, compensation for additional health services and targeted subsidies on HRQoL) found improvements in overall health status measured by the EuroQol 5-Dimension 5-Level (EQ-5D-5L) visual analog scale and by the EQ-5D-5L utility index compared with participants receiving usual care [24]. Similarly, a quasi-experimental trial in rural China among more than 1,400 patients with HTN, testing an integrated care model, coordinating services across village clinics, township, and county hospitals, demonstrated enhanced SF-36 scores—particularly in the physical functioning domain, with no significant improvements observed in patient’s mental health status [25].

In contrast, our intervention resulted in modest improvement in the mental MCS, which was different from previous studies in other in low- and middle-income country settings that have shown significant benefits across both physical and mental domains. For example, a study in Egypt among 1,100 patients with HTN (2017−2019) reported significant improvements in both PCS and MCS scores in the intervention group who received a group-based educational intervention compared to a control group who received usual care [26]. A trial in rural India in 2022 among more than 300 patients with HTN found that an exercise-focused intervention had broad improvements across both physical and psychological domains [27]. A trial in rural China assessing the effectiveness of a mobile health intervention on HRQoL among more than 200 patients with uncontrolled HTN showed that the intervention significantly improved the PCS-12 and MCS-12 scores [28]. These differences may reflect differences in the MCS-12 scores at baseline, intervention content and focus, intensity, delivery mode, or contextual factors such as cultural attitudes toward mental health.

Although our delivered intervention had multiple-components, individual components likely contributed synergistically to the observed improvements in physical HRQoL. While no study has investigated the impact of a storytelling component on HRQoL in adults with uncontrolled HTN, several studies have demonstrated that this intervention can potentially improve HRQoL in patients with other non-communicable chronic diseases. For example, a randomized trial among 60 adults with type 1 diabetes in Iran found that a four-week digital storytelling intervention using peer-created videos and guided reflections significantly reduced diabetes-related distress, burnout, and depressive symptoms, together with improvements in psychological well-being as compared with routine care [29]. Evidence on the impact of home BP monitoring on HRQoL is also limited but promising. For example, a trial among nearly 200 patients with HTN in Honduras and Mexico in 2011 found that automated telephone care management plus home BP monitoring reduced depressive symptoms, medication problems, and improved patient’s general health status and satisfaction with care compared with usual care [30]. While the role and impact of CHWs on patients’ HRQoL has been studied in other chronic conditions such as diabetes [31], little is known about their effect on HRQoL in adults with uncontrolled HTN.

Our multi-component intervention improved HRQoL through strategies that included promoting a healthy lifestyle, increasing medication adherence, and disease self-management strategies. The more substantial improvements in PCS-12 scores suggest that the intervention effectively addressed physical limitations, pain, and general health perceptions in our study population. Components likely contributing to these improvements include enhanced antihypertensive medication adherence, lifestyle modifications such as increased physical activity and healthy dietary changes, improved HTN control, and access to healthcare services.

The relatively modest impact on MCS-12 scores could be explained by several factors. First, our intervention focused on heart health, and not mental health, such as stress management, social support enhancement, or psychological well-being. Second, our study population had a baseline MCS-12 score of 51.9, which was slightly above average, making further improvements in this outcome more difficult to achieve. Third, longer follow-up periods may be necessary to observe meaningful changes in mental health outcomes following physical health improvements. Finally, since the study was conducted during the COVID-19 pandemic, a period marked by significant disruptions to daily life and psychological well-being, this may have impacted the study results, particularly about the mental health outcomes.

### Study strengths and limitations

This study had several strengths, including its well-designed, full-scale cluster-randomized design in multiple communities in a northern rural setting in Vietnam, testing a multi-component intervention, and use of validated measures for assessing HRQoL. Moreover, the intervention was grounded in the existing health system infrastructure and delivered in collaboration with local health authorities, enhancing its feasibility and scalability.

There are several limitations, however, that need to be considered in the context of the present study. These included the study setting in northern Vietnam, which may have resulted in limiting potential generalizability. HRQoL was self-reported, which may have introduced social desirability bias. The follow-up period was relatively short (12 months), potentially limiting the detection of long-term HRQoL changes, particularly in mental health. In addition, we were unable to evaluate each intervention enhancement separately since we examined the holistic impact of a multi-component intervention. Future studies may need to include multiple geographic and urban and rural settings with a longer follow-up period and testing a comprehensive multi-component intervention targeting both physical and mental health for patients with uncontrolled HTN.

### Future intervention directions

Recent literature suggests that integrated approaches that address both physical and psychological aspects of HTN management yield meaningful improvements in HRQoL [25,32]. Given the growing accessibility of digital technologies in Vietnam, future research should explore the implementation of mHealth-based interventions as a feasible and scalable approach to enhance HTN control and improve HRQoL, especially in underserved rural populations.

## Conclusions

This study demonstrated that our multicomponent, community-based intervention improved overall HRQoL among adults with uncontrolled HTN in rural Vietnam, with a greater effect observed in the physical health domain compared to the mental health domain. These findings suggest that our multicomponent strategies are promising approaches to support HTN management in low-resource settings. Although the multi-faceted intervention produced statistically significant improvements in the physical domain, the physical score at 12 months in the intervention group was still relatively low. To achieve more comprehensive improvements in HRQoL and more widespread dissemination, future interventions may need to be intensified and integrate additional components focused on psychological well-being and stress management.

## Supporting information

S1 File CONSORT checklist. (DOCX)
